# Treatment of neuromyelitis optica spectrum disorder: revisiting the complement system and other aspects of pathogenesis

**DOI:** 10.1007/s10354-022-00987-2

**Published:** 2022-12-06

**Authors:** Markus Ponleitner, Paulus Stefan Rommer

**Affiliations:** Department of Neurology, Medical University of Vienna, Waehringer Guertel 18–20, 1090 Vienna, Austria

**Keywords:** Aquaporin 4, Complement system proteins, Therapeutics, Neuroinflammatory diseases, Central nervous system, Aquaporin‑4, Komplementsystemproteine, Therapien, Neuroinflammatorische Erkrankungen, Zentrales Nervensystem

## Abstract

Neuromyelitis optica spectrum disorder (NMOSD) represents a rare neuroimmunological disease causing recurrent attacks and accumulation of permanent disability in affected patients. The discovery of the pathogenic IgG‑1 antibody targeting a water channel expressed in astrocytes, aquaporin 4, constitutes a milestone achievement. Subsequently, multiple pathophysiological aspects of this distinct disease entity have been investigated. Demyelinating lesions and axonal damage ensue from autoantibodies targeting an astroglial epitope. This conundrum has been addressed in the current disease model, where activation of the complement system as well as B cells and interleukin 6 (IL-6) emerged as key contributors. It is the aim of this review to address these factors in light of novel treatment compounds which reflect these pathophysiological concepts in aiming for attack prevention, thus reducing disease burden in patients with NMOSD.

## Abstract (de)

Bei der Neuromyelitis-optica-Spektrum-Erkrankung („neuromyelitis optica spectrum disorder“, NMOSD) handelt es sich um eine seltene neuroimmunologische Erkrankung, die bei den betroffenen Patient*innen zu wiederkehrenden Schüben und bleibender Behinderungen führt. Nach der bedeutsamen Entdeckung des pathogenen Immunglobulin-G1(IgG1)-Antikörpers, dessen Ziel aus einem in Astrozyten exprimierten Wasserkanal, Aquaporin‑4, besteht, wurden zahlreiche pathophysiologische Aspekte dieser Krankheitsentität untersucht. Trotz des astroglialen Epitops sind die Folgen dieser Aquaporin-4-IgG-Autoantikörper demyelinisierende Läsionen und axonale Schäden. Im aktuellen Krankheitsmodell stehen die Aktivierung des Komplementsystems sowie B‑Zellen und Interleukin‑6 (IL-6) als Schlüsselfaktoren im Vordergrund. Ziel der vorliegenden Übersichtsarbeit ist es, diese Faktoren im Hinblick auf neuartige Behandlungspräparate zu untersuchen. Diese setzen an den pathophysiologischen Schnittstellen an, um Schübe zu verhindern und so die Krankheitslast bei Patient*innen mit NMOSD zu verringern.

## Introduction

The first description of a disease primarily affecting the optical nerves and the spinal cord—then Devic’s disease—was published over 100 years ago [1]. The term *neuromyelitis optica* (NMO) was coined based on the hallmark symptoms still included in modern diagnostic criteria [2]. The distinction between the more common Multiple Sclerosis (MS), which shares some clinical and radiological disease characteristics [3], and NMO depended solely on clinical features until the antibody against the astroglial surface protein aquaporin 4 (AQP4-IgG), highly specific for NMO, was discovered in 2004 [4, 5]. With this diagnostic tool, the phenotype was expanded, resulting in the novel term *neuromyelitis optica spectrum disorder *(NMOSD) [6]. The prevalence of AQP4-IgG in patients diagnosed with NMOSD according to the international Panel for NMO Diagnosis (IPND) criteria has been shown to be 73–90% [7, 8]. Investigation of patients fulfilling diagnostic criteria for NMOSD but seronegative for AQP4-IgG eventually led to the discovery of antibodies targeting myelin oligodendrocyte glycoprotein (MOG-IgG).

One study found that 73% of 132 patients fulfilling the IPND criteria had AQP4-IgG antibodies; of the AQP4-IgG-seronegative patients, 42% had antibodies targeting MOG (11% of patients fulfilling IPND criteria) and the remaining patients were classified as double seronegative. None of the patients with AQP4-IgG were definitely positive for MOG-AB and vice versa [9], indicating that these antibodies are mutually exclusive. This was also reflected in pathological analyses [10]. Most evidence concerning pathophysiology, treatment strategies, and therapeutic agents is based on AQP4-IgG-seropositive patients. The detailed nature of double-seronegative (AQP4- and MOG-AB-negative) NMOSD requires additional research.

With the emergence of the pathogenic antibody, pathophysiological disease models were conceptualized, which are the basis of novel treatment options in NMOSD. It is the aim of this review to relate these models to their modes of action, paying special attention to the complement system.

## Pathogenesis

The discovery of AQP4-IgG marked an important milestone in NMOSD research in that the origin of central nervous system (CNS) lesions appears to be an astrocytopathy [4]. AQP4 constitutes the most abundant water channel in the CNS. It is found on astrocytes, with the highest expression on their foot processes, which are an integral part of the blood–brain barrier [11, 12]. Additionally, AQP4 is expressed in the glia limitans and ependyma [13].

AQP4-IgGs in NMOSD are primarily of the IgG1 subclass. This indicates that a subclass switch through interaction with autoreactive CD4+ T cells must have occurred. Antibody production occurs predominantly in plasma cells outside of the CNS, which is reflected in 500-fold higher titers in serum compared to CNS [14, 15]. These cells can be further characterized as CD19^int^, CD27^+^, CD38^+^, and CD180^−^, indicating a plasmablast phenotype [16]. Antibody production and plasmablast survival crucially depend on interleukin 6 (IL-6), which, in fact, is markedly increased in the serum and cerebrospinal fluid (CSF) of patients with NMOSD in comparison to healthy controls and, importantly, patients with MS [17, 18]. In vitro studies have shown that the IL‑6 receptor (IL-6R), is highly expressed on the plasmablast fraction in question. Additionally, antibody production and plasmablast survival were directly correlated with IL‑6 levels, while blockage of the IL-6R reduced both metrics [15].

Antigen recognition occurs through disruption of the blood–brain barrier (BBB) or at sites with high BBB-permeability (e.g., circumventricular organs, i.e., area postrema).

Binding of AQP4-IgGs induces complement activation and subsequent cell lysis of astrocytes via complement-dependent cytotoxicity (CDC). Cell death and complement activation releases pro-inflammatory mediators, resulting in recruitment of T and B cells as well as mono- and granulocytes and some eosinophils. This immune reaction induces antibody-dependent cellular cytotoxicity (ADCC), demyelination, and tissue damage including axonopathy [19, 20].

An alternative explanation for demyelination involves secondary damage to oligodendrocytes without astrocyte necrosis: it has been shown that expression of the astrocytic glutamate transporter 1 (GLT‑1; excitatory amino acid transporter 2 [EAAT-2]) requires co-expression of AQP4 [21]. Co-internalization of GLT‑1 upon AQP4 antibody binding likely results in glutamate-induced cytotoxicity, causing oligodendrocyte damage and subsequent demyelination in the nearby environment without astrocyte necrosis [22]. Thus, one can envision two distinct reactions following antigen recognition by AQP4-IgG: (i) degradation and complement activation causing astrocyte lysis and severe tissue damage with necrotic lesion formation and (ii) internalization of AQP4 causing primary oligodendropathy and solely demyelination lesions [23, 24]. These concepts are in line with different lesion types described in patients suffering from NMOSD [19, 20].

### The complement system revisited

The complex complement system is part of the innate humoral immunity in humans and has emerged as a key player in the pathophysiology of several autoimmune diseases, including NMOSD.

Since a first description of particles complementing the immune system by Paul Ehrlich [11], a system of more than 30 proteins has been discovered and termed *the complement system*. Deficiencies of these complement factors, acquired or hereditary, have been associated with increased susceptibility to infections or manifestation of autoimmune disorders, underlining the importance of this integral part of our immune system [12].

Once activated by one of three known pathways (classical, lectin, alternate), the complement cascade undergoes positive feedback propagation, converging towards the activation of what is denoted the membrane attack complex (MAC). The MAC comprises oligomerized C9 subunits attached to preformed complement structures, which gives rise to a pore that is forced through the target cell membrane, resulting in lysis and cell death.

The broadly accepted role of the pathogenic AQP4-IgG1 antibody in NMOSD renders the classical pathway most relevant in this disease. Upon epitope recognition, a small conformational change in the IgG1, IgG‑2, and IgG‑3 subclasses (not IgG4) allows for binding of C1q. The following cascade results in assembly of C3 convertase, which cleaves C3 into C3a, an anaphylatoxin, and C3b. Incorporation of the latter into the pre-existing C3 convertases continues to form a C5 convertase [13].

C5 convertase activity yields C5a, another potent inflammatory chemokine, and C5b, the foundation of the MAC. C5b co-aggregates with C6, C7, and C8, forming an increasingly stable tetrameric complex anchored to the surface membrane, which finally allows for association of the MAC from 10–16 C9 molecules, piercing the membrane and causing lysis [14, 15].

## Epidemiology

Several nationwide studies found the prevalence of NMOSD to range from 0.37–10/100,000 (highest prevalence among African, East Asian, and Latin American populations), predominantly manifesting as a relapsing disease (90–99%) with a documented predilection in females with a ratio of approximately 5:1 to 10:1 [16–19]. Median disease onset typically occurs between age 30 and 40, while onset in children and older patients has been described [3].

## Clinical manifestations

The term neuromyelitis optica, as the disease was previously known, reflects two of the most characteristic manifestations of relapses in patients afflicted by this condition: (i) bilateral or rapidly sequential severe optic neuritis (ON) and (ii) longitudinally extensive transverse myelitis (LETM; extending over at least three vertebrae) [6].

In contrast to MS, the relapses are often much more severe, so that significant disability may remain even after a first relapse [20].

In addition to these classical manifestations, patients have been reported with insatiable singultus, nausea, and vomiting (lesion site: area postrema). Upon involvement of the brainstem and cervical spinal cord, symptoms include respiratory insufficiency, cardiac arrhythmias, dysphagia, dizziness, and oculomotor disturbances [3, 21].

Lesions in the diencephalon have been associated with cases of narcolepsy, hypopituitarism with correspondingly impaired hormone secretion (including antidiuretic hormone), and temperature regulation disorders [22, 23]. Posterior reversible encephalopathy syndrome (PRES), aphasia, apraxia, seizures, and confusion have been reported with corresponding lesions in the cerebrum (cerebral syndrome) [24, 25].

The disease course of NMOSD manifests in attacks, while progressive courses have not been described [26]. MRI data, however, seem to show an accumulation of “silent” lesions, without corresponding clinical manifestations. Depending on the symptomatology, patients suffer from marked disabilities, sometimes even life-threatening situations (respiratory failure) [3]. During pregnancy, relapses (unlike MS) are not uncommon [27].

In NMOSD, other autoimmune diseases (e.g., systemic lupus erythematosus or myasthenia gravis) are also not uncommon [28].

### Outcome prediction

A large multicenter dataset of 441 patients who collectively experienced 1976 attacks was investigated to extract relapse likelihood and disability. Among other things, according to this model, female rather than male patients have a higher risk for myelitis and overall attacks. The risk of acquiring permanent disability was higher for female patients. Younger age (≤ 35 years) at initial relapse was associated with a higher risk for optic neuritis attacks and permanent visual impairment [20].

## Diagnosis and differential diagnosis

The latest revision of the diagnostic criteria (International Panel of NMO Diagnosis, IPND) features six core clinical symptoms including optic neuritis, acute myelitis, area postrema syndrome, acute brainstem syndrome, symptomatic narcolepsy, or acute diencephalic syndrome with typical MRI lesions and symptomatic cerebral syndrome with typical MRI lesions.

None of these symptoms are disease specific, however, which is why potential differential diagnoses, the patient’s aquaporin-4-antibody (AQP4-IgG) status, and MRI data must be considered to confirm the diagnosis of NMOSD [2].

While AQP4-IgGs are detectable in most patients, a seronegative phenotype (double negative for AQP4- and MOG-IgGs) has been described, which requires more stringent clinical and MRI criteria to establish a diagnosis of seronegative NMOSD [2].

NMOSD-associated relapses cause accumulation of permanent disabilities, oftentimes within a short disease duration and independent of the presence of AQP4 antibody status and titer [3, 29]. Additionally, cloud-like enhancement in MRI appears to be specific for NMOSD [30].

### Diagnostic approach

While one core clinical criterion is required for cases with evidence of AQP4-IgG, two core criteria are required for cases without the specific antibodies (seronegative), one of which must be optic neuritis, myelitis, or area postrema syndrome. In seronegative cases, additional MRI criteria (noted in parentheses) must be followed. The core criteria are:

1. Optic neuritis (MRI criterion: unremarkable or nonspecific MRI of the cranium, or T2 hyperintensity of the optic nerve, or contrast radiography of at least half the length of the optic nerve or chiasm).
2. Myelitis (MRI criterion: intramedullary lesions or spinal atrophy with extension over three vertebral segments).
3. Area postrema syndrome including singultus, nausea, or vomiting not otherwise explicable (MRI criterion: lesion in the dorsal medulla oblongata/area postrema).
4. Acute brainstem syndrome (MRI: peri-ependymal brainstem lesion).
5. Symptomatic narcolepsy or diencephalic syndrome with corresponding evidence of a diencephalic lesion.
6. Symptomatic cerebral syndrome with corresponding lesions (e.g., thalamus, corticospinal tract).

Red flags (e.g., progressive course, paraparesis within less than 4 h or continuous clinical worsening over 4 weeks, persistent gadolinium uptake in the spine) should raise concerns about alternative diagnoses.

An important differential diagnosis to NMOSD is constituted by MOG encephalomyelitis (MOG-EM) or MOG antibody-associated autoimmune disorders (MOG-AD); this distinct disease was defined after the discovery of MOG-IgG [2, 31–34]. The clinical and radiological phenotype of MOG-AD partly overlaps with NMOSD and acute disseminated encephalomyelitis (ADEM). Thus, MOG-AD constitutes an important differential diagnosis in suspected demyelinating disease [2, 32, 35]. In MOG-AD, lesions are predominantly found intracortically [10]. In contrast to the primarily astrocytopathic NMOSD, however, the pathophysiology of MOG-AD depends on antibody-mediated damage to tissue expressing MOG: the outer layers of the myelin sheath and oligodendroglia [36–39]. Diagnostic criteria essentially depend on (i) the presence of IgG antibodies targeting MOG with (ii) characteristic neurological symptoms (including optic neuritis, myelitis, brainstem encephalitis, and encephalitis) resulting from (iii) demyelination. Also (iv), the presence of any red flags (e.g., sudden symptom onset, chronic disease progression, MRI lesion configuration or CSF results suggestive of MS or another inflammatory CNS disease, low or borderline MOG-IgG titers with possible other etiology) should prompt reevaluation of the diagnosis [33, 34, 40].

Tables 1 and 2 summarize the diagnostic approach and the diagnostic criteria.

**Table 1:** Diagnostic criteria depending on antibody status (AQP4-IgG)

Seropositive	Seronegative (“double negative”)
≥ 1 of 6 core clinical criteria	≥ 2 of 6 core clinical criteria attributable to ≥ 1 relapses
	≥ 1 core criterion must be either of – optic neuritis – acute myelitis as defined by LETM – area postrema syndrome (e.g., singultus not otherwise explained nausea)
	Dissemination in space must be met (≥ 2 core clinical criteria)—additional MR criteria should be met, if applicable
Positive AQP4-IgG status	Negative test for AQP4-IgG with the best available test
Exclusion of alternative diagnoses	Exclusion of alternative diagnoses

*LETM* longitudinally extensive transverse myelitis, *AQP4-IgG* aquaporin-4 immunoglobulin G, *MR* magnetic resonance

**Table 2:** Diagnostic criteria in NMOSD

Core criteria	MRI additional criteria
Optic neuritis	*Acute optic neuritis:* requires a) unremarkable cranial MRI or nonspecific white matter changes or b) T2 hyperintense lesions or gadolinium-enhancing lesion of at least half of the optic nerve or chiasm
Acute myelitis	*Acute myelitis:* requires lesion intramedullary over three vertebral segments or atrophy extending over three vertebral segments in patients with history of acute myelitis
Area postrema syndrome: episode of otherwise unexplained singultus, nausea, or vomiting	*Area postrema syndrome:* requires a lesion located dorsally in the medulla oblongata or in the area postrema
Acute brainstem syndrome	*Acute brainstem syndrome:* requires a periependymal brainstem lesion
Symptomatic narcolepsy or acute diencephalic syndrome with NMOSD-typical diencephalic changes on MRI	–
Symptomatic cerebral symptoms in combination with MRI lesions typical for NMOSD	–

*MRI* magnetic resonance imaging, *NMOSD* neuromyelitis optica spectrum disorder

## Therapy

### Acute attack therapy

Therapeutic approaches to acute attacks include high-dose intravenous methylprednisolone (HDMP; 1000 mg methylprednisolone per day for 5 consecutive days) and apheresis (plasma exchange [PLEX] or immunadsorption [IAS], 5–7 cycles). The efficacy of timely initiation of HDMP, especially for optic neuritis in NMOSD [41], has been shown in several studies [29, 42, 43].

AQP4 antibody titers have been shown to correlate with the course of the disease in some patients and high antibody serum levels have been associated with attacks [44]. Thus, early therapeutic apheresis has been suggested as an effective add-on or even first-line therapy, especially in the case of a previous good response to apheresis, myelitis, severe attacks (EDSS ≥ 4 or visual acuity < 20/100), or insufficient response to HDMP [29, 45–56]. So far, apheresis therapies (PLEX, IAS) have shown similar efficacy, while plasma exchange has been far more extensively studied [46].

In a retrospective study investigating 83 NMOSD attacks in 59 patients, the combination of PLEX and HDMP resulted in a higher remission rate (65%) than HDMP alone (35%) [45]. If both approaches (HDMP and PLEX) are used concomitantly, on days with PLEX, HDMP should be administered after rather than before plasma exchange [57, 58].

### Attack prevention

Over the predominantly relapsing disease course of NMOSD, frequent severe attacks cause accumulation of permanent disability. Hence, intensive attack-prevention therapy remains paramount. This is also true for patients aged > 50 years, who appear more prone to retaining persistent deficits after attacks [29, 59–61]. Importantly, attack-prevention therapy should be started following the onset attack, as this accounts for only 25% of permanent disability in patients with NMOSD, thus highlighting the potential for preventing further disability [20].

Oral continuation of steroids following HDMP after attacks to bridge the time to full treatment effect (up to several months) has been recommended, although high-quality evidence is still lacking. MS must be ruled out in patients with suspected NMOSD, as several of the therapies used in MS appear to be ineffective or even harmful in NMOSD. This has been indicated in different studies for β‑interferon, glatiramer acetate, natalizumab, fingolimod, dimethyl fumarate, and alemtuzumab [62–68].

#### Conventional immunosuppressive therapies

Commonly used off-label treatments comprise azathioprine (AZA), an inhibitor of purine synthesis [69], which showed efficacy in reducing the annualized relapse rate (ARR) and stabilizing the EDSS (expanded disability status scale) in patients with NMOSD [70–76]. A randomized-controlled, open-label single-center study comparing the efficacy of AZA with adjunctive oral glucocorticoid therapy to rituximab (RTX) showed that RTX was significantly more effective [77]. A similar efficacy to AZA in the treatment of NMOSD has been reported for mycophenolate mofetil (MMF; an inhibitor of guanidine synthesis), the folate antagonist methotrexate (MTX), as well as the intercalating agent and inhibitor of topoisomerase II mitoxantrone (MTN; MTX and MTN in combination with oral glucocorticoid therapy) [78–82].

Limited evidence based on reports of individual cases suggests effectiveness of intermittent PLEX in long-term attack prevention [83].

**Fig. 1:**
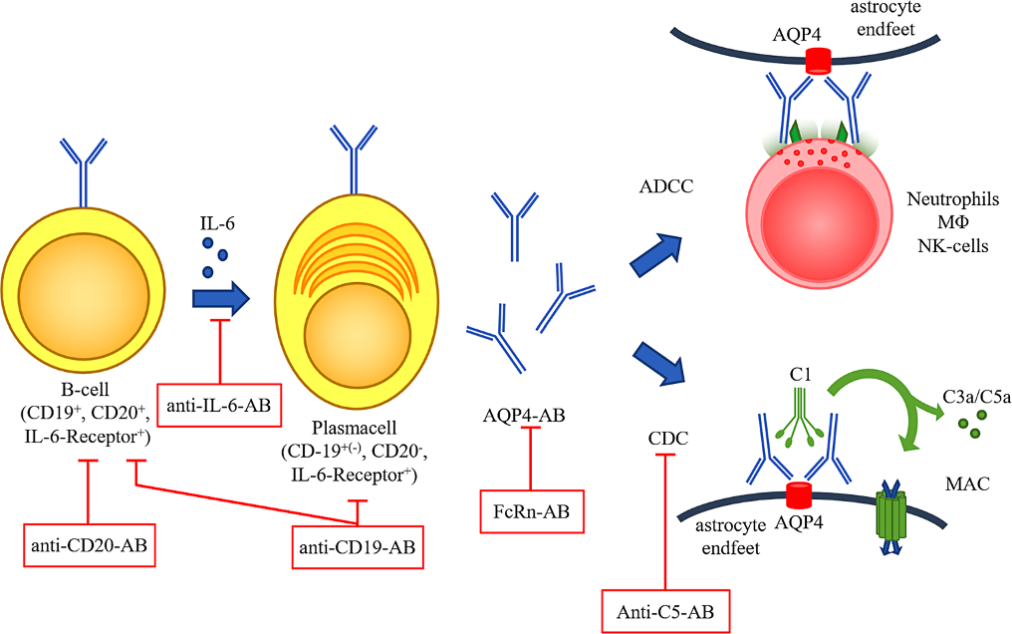
Pathophysiology and treatment options in NMOSD: production of pathogenic AQP4-IgGs occurs in plasmablasts, which derive from B cells upon IL-6 stimulation. Upon antigen recognition, damage occurs mainly through two pathways: i: complement dependent cytotoxicity (CDC): antigen-bound AQP4-IgGs trigger the classical pathway for complement activation, resulting in binding and activation of C1. The downstream cascade releases C3a and C5a, potent pro-inflammatory chemokines and forms a tetrameric anchor (C5b, C6, C7, C8) to the surface membrane (not shown). This provides the scaffold for association of several C9 molecules. These aggregate to form a pore, which is finally inserted into the membrane causing cell lysis. ii: antibody dependent cellular cytotoxicity (ADCC): antigen-bound AQP4-IgGs bind to Fc-receptors expressed on the surface of neutrophils, macrophages (MΦ) and NK cells, causing necrosis of the target cell as exemplified by release of cytotoxic mediators from NK-cell granules.The modern armamentarium of therapies interacts in many crucial points in the pathophysiological cascade, as indicated with the *red bars*. *CD* cluster of differentiation, *AB* antibody, *IL‑6* interleukin 6, *AQP4‑AB* aquaporin 4 antibody, *FcRn* neonatal Fc-Receptor, *ADCC* antibody dependent cellular cytotoxicity, *CDC* complement dependent cytotoxicity, *MAC* membrane attack complex, *NK cells* natural-killer cells

Recently, three treatments targeting the IL‑6 receptor, CD19 on B and plasma cells, or inhibiting the complement system have been approved for the treatment of AQP4-IgG-positive NMOSD patients by the United States of America Food and Drug Administration (FDA) and the European Medicines Agency (EMA). Fig. 1 illustrates the different pathophysiological principles and mechanism of action in NMOSD.

#### B cell-depleting therapies

Rituximab (RTX), a B cell-depleting chimeric monoclonal antibody targeting CD20 commonly used off label in NMOSD, has shown good efficacy for reduction of ARR, disease progression, and disability. Depending on the reported dosing regimen, rituximab therapy was usually initiated by infusion of 1 g or 375 mg/m^2^ intravenously, repeated after 2 weeks with continuous biannual infusions (every 6 months) of 1 g or 375 mg/m^2^ for maintenance therapy; pre-treatment with antihistamines, antipyretics, and steroids, as well as close patient monitoring are required to minimize acute infusion reactions (AIRs) [77, 84–89]. Although rituximab therapy in different patient cohorts was associated with adverse events (AEs) including AIRs, cardiac events, infections, hypogammaglobulinemia (associated with more frequent and more severe infections), and others [90–93], observations in NMOSD patients revealed excellent efficacy and acceptable safety [94, 95].

Inebilizumab is another treatment option in NMOSD recently approved in the United States [96] and Europe [97]. This humanized monoclonal antibody binds to CD19-positive cells and causes depletion of the specific B cell subset plasmablast pool associated with peripheral AQP4-IgG production.

In contrast to mature plasma cells, NMOSD patients have shown expansion of plasmablasts still expressing CD19. Thus, targeting of this epitope directly interferes with the synthesis of pathogenic AQP4-IgG.

Accordingly, inebilizumab exhibited good efficacy and safety in the phase II/III N‑MOmentum trial (*n* = 230) versus placebo [98]; due to the recency of approval, however, long-term data are still missing. Treatment initiation features two intravenous doses of 300 mg in weeks 0 and 2. Subsequently, infusions of 300 mg of inebilizumab every 6 months constitute maintenance therapy. Similar to rituximab, the strategy for minimizing AIRs consists of pre-treatment with antihistamines, antipyretics, and steroids, as well as close patient monitoring. The most common adverse reactions reported in the N‑MOmentum trial were urinary tract infections, headache, arthralgia, nausea, and back pain. As with RTX, B cell depletion by inebilizumab may cause hypogammaglobulinemia, associated with an increased rate of opportunistic infections. Hence, regular laboratory studies are recommended [98].

#### IL-6-inhibiting therapies

The effect of satralizumab, a humanized monoclonal antibody against the interleukin‑6 (IL-6) receptor, in the treatment of AQP4-IgG-seropositive and seronegative NMOSD has been studied in two randomized-controlled, double-blind phase III trials (SAkuraSky, SakuraStar). Results showed a good efficacy in the study group compared with placebo as an add-on to baseline immunosuppressive therapy [99] and as monotherapy [100]. Subgroup analysis indicated a marked reduction of relapse risk in patients with AQP4-IgG-positive NMOSD compared with placebo, in keeping with the requirement of IL‑6 stimulation for antibody production and plasmablast survival [101].

In patients with AQP4-IgG-negative NMOSD, however, this effect could not be observed [99, 100].

Considering the lower number of seronegative patients—reflecting the reported distribution among the NMOSD patient population—neither study was powered nor intended to analyze effects in this subgroup, which is why further investigation is required before a final statement is possible.

Satralizumab was approved for treatment of AQP4-IgG-positive NMOSD by the FDA and the EMA [102, 103]. The therapy is administered as a subcutaneous injection of 120 mg of satralizumab at weeks 0, 2, and 4, with subsequent injection every 4 weeks. Satralizumab exhibited a favorable risk profile with similar rates of AEs, SAEs, infections, and serious infections per 100 patient years in treatment and placebo groups, respectively [99, 100].

Tocilizumab, another humanized monoclonal antibody targeting the IL‑6 receptor frequently used in rheumatic diseases, has shown a promising effect in clinical disability and reduction of relapse rate in highly relapsing, treatment-resistant NMOSD in some case reports and small case series as an off-label treatment option [104–110]. In a phase II, randomized, open-label trial (TANGO, *n* = 118), intravenous tocilizumab showed a more pronounced reduction of relapse risk, especially in the subgroup of patients also suffering from other autoimmune disorders, than azathioprine; both treatment options exhibited a similar safety profile [111]. Among studies in NMOSD patients, intravenous application of 8 mg/kg every 4 weeks has been more common, while reports on subcutaneous administration also exist [104–111].

#### Therapies interfering with the complement system

Based on the pathogenesis with activation of the complement system and subsequent CDC, therapies interfering with the complement system have been approved or studied in NMOSD:

Eculizumab, a humanized monoclonal antibody binding to the C5 complement element and thus inhibiting formation of the membrane attack complex (MAC), has shown excellent results in the therapy of AQP4-IgG-positive, highly active, relapsing NMOSD [112, 113]. A recent interim analysis of the open-label extension of the PREVENT study confirmed the outstanding efficacy in terms of ARR reduction (0.025 in the treatment group vs. 0.35 in the placebo group); the vast majority of treated patients at 192 weeks had remained entirely relapse free. Rates of AEs and serious adverse events (SAEs) in the study group were comparable to the PREVENT placebo group, while the rate of serious infections was lower in the treatment group compared to placebo [114]. A single patient treated with eculizumab and azathioprine died from pulmonary empyema [113]. Infections with *Neisseria meningitidis*, as described in previous studies evaluating eculizumab in the treatment of other diseases, were not reported; a vaccination against this pathogen is required for eculizumab treatment [113–115]. The treatment regimen of eculizumab consists of four infusions of 900 mg once per week over 4 weeks (induction phase) and infusions of 1200 mg every 14 ± 2 days thereafter (maintenance phase); pre-treatment is not required. In the light of these data, eculizumab has been approved for treatment of adult patients with AQP4-IgG-positive NMOSD in Europe [116] and the United States [117], among other countries worldwide.

#### Treatments in development

##### Neonatal Fc receptor

It has been shown that the increased half-life of IgG compared to other immunoglobulin subclasses relies on interaction with the neonatal crystallizable fragment (Fc) receptor (FcRn). Binding of internalized IgG to FcRn results in recycling and release at the cell surface, rather than lysosomal degradation [118, 119].

These insights into the mechanism of antibody metabolism elucidated the crucial role of FcRn (neonatal Fc receptor), which may provide a novel approach for a biological long-term immune therapy. In fact, the monoclonal IgG2 antibody satralizumab employs this technique, enabling antibody recycling with increased half-life and dosing every 4 weeks [99, 100]. Additionally, ravulizumab, a novel anti-C5 monoclonal antibody, also utilizes this strategy, allowing dosing every 8 weeks compared to the dosing every 2 weeks known from eculizumab. It was shown to be non-inferior in the treatment of paroxysmal nocturnal hemoglobinuria [120].

Furthermore, a human monoclonal antibody (HBM9161) targeting the FcRn to reduce systemic IgG half-life, including that of the pathogenic AQP4-IgG, is currently under development [121].

##### Stem cell transplantation

A recent meta-analysis of the available literature showed excellent safety and efficacy in terms of progression-free survival of patients treated with autologous hematopoietic stem cell transplantation in NMOSD [122]. This warrants additional evaluation in future studies to research the auspicious goal of developing a treatment offering a potential cure for this rare, yet severe disease.

### Therapy discontinuation

Discontinuation of long-term attack-prevention therapy after an extensive attack-free period, while not extensively investigated (17 patients treated with AZA, MMF, RTX, MTX followed by MMF), has resulted in resurgence of attacks within a few months in most cases [123]. The longest interval of relapse-free periods was observed after discontinuation of RTX therapy [124]. Hence, when or whether to terminate effective long-term treatment remains a challenging question.

## Conclusion

Neuromyelitis optica spectrum disorders (NMOSD) constitute a rare neuroimmunological disease with a high burden of permanent disability following severe attacks that primarily occurs in adults. In the past decades, several milestones have been achieved in researching this distinct condition, which was initially thought to be a severe sub-form of multiple sclerosis. The discovery of highly specific, pathogenic AQP4-IgGs in 2004 has given rise to a pathophysiological model hinged on the astroglial epitope aquaporin 4 as the target epitope and origin of lesion formation after recognition by pathogenic antibodies.

Treatment strategies in NMOSD are in keeping with pathophysiological considerations:

1. *Targeting B and plasma cells*
  NMOSD pathogenesis depends on effects of the highly specific AQP4-IgG. Thus, elimination of antibody production has been shown to be an efficacious strategy for reducing relapses and preventing relapse-associated permanent disability. From a pathophysiological standpoint, targeting CD19 rather than CD20 seems superior, since antibody-producing plasmablasts in NMOSD have been shown to express this marker while being CD20 negative, thus broadening the effect on the source of the pathogenic AQP4-AB.
2. *Targeting pro-inflammatory mediators*
  Since plasmablast survival and antigen production are dependent on elevated IL‑6 with high IL-6R expression, the removal of this vital inflammatory stimulus has shown promising treatment success leading to the approval of satralizumab, which is now available as on-label treatment in seropositive patients with NMOSD.
3. *Targeting pathogenic antibodies*
  Direct removal of pathogenic antibodies via plasmapheresis or immunoadsorption has emerged as a viable treatment strategy of acute attacks, hinging on the pivotal role of AQP4-IgG in NMOSD, with some studies already reporting improved efficacy of combination therapy (HDMP + PLEX) compared to HDMP monotherapy.
  Additionally, the increasing attention on the FcRn pathway as an essential aspect in IgG metabolism has provided novel modes of action in the treatment of NMOSD. Depletion of pathogenic antibodies by denying recycling and significant reduction in half-life on the one hand and increase of dosing intervals of therapeutic antibodies employing the FcRn pathway on the other was achieved through application of this mechanism.
4. *Targeting the complement system*
  Based on current pathophysiological models, complement activation mediates lesion formation by astrocyte-targeted CDC prior to leukocyte infiltration, demyelination, and axon damage. Thus, inhibition of C5 cleavage to C5a, a potent anaphylatoxin, and C5b, the first step of MAC formation, should have beneficial effects on the disease course. Although affecting only the final steps of the complement cascade, this has been reflected in the outstanding efficacy of eculizumab, with subsequent approval in AQP4-seropositive NMOSD.
  Interference with the complement system, however, requires heightened vigilance for infections with gram-negative bacteria, e.g., meningococcal meningitis.

## Abbreviations

ADCC: Antibody-dependent cellular cytotoxicity
ADEM: Acute disseminated encephalomyelitis
AIR: Acute infusion reaction
AQP4: Aquaporin 4
ARR: Annualized relapse rate
AZA: Azathioprine
BBB: Blood–brain barrier
CD: Cluster of differentiation
CDC: Complement-dependent cytotoxicity
EAAT‑2: Excitatory amino acid transporter 2
EDSS: Expanded disability status scale
Fc: Crystallizable fragment
FcRn: Neonatal Fc receptor
GLT‑1: Glutamate transporter 1
HDMP: High-dose methylprednisolone
IAS: Immunadsorption
Ig: Immunoglobulin
IL: Interleukin
IPND: International Panel for NMO Diagnosis
LETM: Longitudinally extensive transverse myelitis
MAC: Membrane attack complex
MMF: Mycophenolate mofetil
MOG-AD: Myelin oligodendrocyte glycoprotein antibody associated autoimmune disorder
MRI: Magnetic resonance imaging
MS: Multiple sclerosis
MTN: Mitoxantrone
MTX: Methotrexate
NMO: Neuromyelitis optica
NMOSD: Neuromyelitis optica spectrum disorder
PLEX: Plasma exchange
PRES: Posterior reversible encephalopathy syndrome
RTX: Rituximab
